# Temporal variability of a protected multispecific tropical seagrass meadow in response to environmental change

**DOI:** 10.1007/s10661-019-7977-z

**Published:** 2019-11-26

**Authors:** E. Alonso Aller, J. S. Eklöf, M. Gullström, U. Kloiber, H. W. Linderholm, L. M. Nordlund

**Affiliations:** 1 Department of Ecology, Environment and Plant Sciences, Stockholm University, Stockholm, Sweden; 2 Department of Biological and Environmental Sciences, University of Gothenburg, Kristineberg, Fiskebäckskil, Sweden; 3 Chumbe Island Coral Park (CHICOP), Zanzibar, Tanzania; 4 Regional Climate Group, Department of Earth Sciences, Gothenburg University, Gothenburg, Sweden; 5 Natural Resources and Sustainable Development, Department of Earth Sciences, Campus Gotland, Uppsala University, Uppsala, Sweden

**Keywords:** Monitoring, Temporal dynamics, Marine protected area, East Africa, Tanzania

## Abstract

In a changing environment, there is an increasing interest to monitor ecosystems to understand their responses to environmental change. Seagrass meadows are highly important ecosystems that are under constant pressure from human activities and climate impacts, with marked declines observed worldwide. Despite increasing efforts, monitoring of multispecific tropical seagrass meadows is scarce, particularly in low-income regions. Based on data from a monitoring programme in a marine protected area in Zanzibar (Tanzania), we assessed temporal changes in seagrass cover and species composition during a 10-year period in relation to local variability in environmental variables. We observed a strong, gradual decline in seagrass cover and changes in species composition, followed by a period of recovery. However, the timing and length of these temporal patterns varied in space (between transects). Multiple environmental variables—cloud cover, temperature, storm occurrence, sunspot activity, and tidal amplitude and height—influenced seagrass cover, although only to a minor extent, suggesting that the monitored seagrass meadow may be influenced by other unmeasured factors (e.g. water currents and sediment movement). Our results show that seagrass meadows can be highly dynamic at small (10–50 m) spatial scales, even in the absence of major local anthropogenic impacts. Our findings suggest that high-resolution monitoring programmes can be highly valuable for the detection of temporal changes in multispecific seagrass meadows; however, to understand the causes of change, there is a need of long-term (> 10 years) data series that include direct measurements of environmental variables and extreme events.

**Electronic supplementary material:**

The online version of this article (10.1007/s10661-019-7977-z) contains supplementary material, which is available to authorized users.

## Introduction

In a time of rapid environmental changes, there has been an increasing interest to monitor ecosystems and understand the causes of gradual as well as sudden ecosystem changes (Callahan 1984). Moreover, detecting ecosystem change is imperative for successful natural resource management, where interventions can be seen as ‘experiments’ whose outcomes must be thoroughly evaluated (Holling 1978). Terrestrial environmental monitoring dates back several centuries, but for long lagged in the marine environment. Over the last few decades, there has been a dramatic increase in efforts to assess how marine ecosystems change over time and respond to environmental changes, particularly in the coastal zone.

Seagrass meadows are one of the most important coastal habitats occupying inter- and subtidal nearshore environments and are frequently emphasized in coastal ecosystem health assessments (Borum et al. 2004; Romero et al. 2015; Roca et al. 2016). Even though there are only about 60 species of seagrass globally (Short et al. 2001), they occur along all continents except Antarctica (Green and Short 2003). Seagrasses are ecosystem engineers that, by buffering wave action, stabilizing sediments, and creating structurally complex and highly productive habitats for other organisms, form one of the most productive ecosystems on Earth (Duarte and Chiscano 1999). By existing at the border between land and sea, and generally being relatively accessible to humans, seagrasses provide many ecosystem services, including fisheries, carbon sequestration, and water purification (Koch et al. 2006; Duarte et al. 2013; Nordlund et al. 2016). Most seagrass meadows are monospecific (with a single seagrass species) but multispecific meadows are common in certain regions, particularly in the tropics, where overall seagrass diversity is the highest.

Seagrass meadows are naturally dynamic in terms of seagrass cover and species composition, varying greatly at seasonal (intra-annual) to centennial time scales (Duarte et al. 2006). Moreover, seagrass meadows are under increasing pressure from human activities, ranging from local physical disturbances to global climate change, resulting in a globally accelerating rate of seagrass loss since the 1950s (Waycott et al. 2009). Over the last decades, there has been an increasing understanding that global climate change, in addition to local disturbances, presents a potential major threat to seagrasses. Even though most seagrasses are relatively tolerant to moderate and gradual climatic changes, extreme events such as heatwaves (Campbell et al. 2006), severe storms (Preen et al. 1995; Short et al. 2016), extreme rain events (Björk et al. 1997; Short et al. 2016), extreme low-tides (causing leaf desiccation; Björk et al. 1999), and extreme low-tide water temperatures (George et al. 2018), can strongly impact seagrasses. While the number of monitoring programmes focused on tropical multispecific seagrass meadows has increased over the last decade, to date, most long-term programmes have been conducted in monospecific meadows in temperate areas (e.g. Bernard et al. 2007; Ball et al. 2014; Shelton et al. 2016). Thus, we have a limited knowledge about the extent to which these impacts are species-specific and whether response diversity (that different, co-occurring species respond in different ways to a shared stressor, Elmqvist et al. 2003) in multispecific meadows can provide a basis for an insurance effect of high seagrass diversity.

In temperate regions, particularly Europe and the USA, but also temperate Australia, seagrass meadows are regularly included in annual monitoring programmes of shallow-water submerged aquatic vegetation. There are also a number of specific seagrass monitoring programmes that have helped shed light on the impacts of environmental change on seagrass dynamics (e.g. Marbà and Duarte 1997; Ball et al. 2014; Shelton et al. 2016; Lefcheck et al. 2017). Seagrasses typically respond markedly to environmental degradation (e.g. low water quality), and thus they can be seen as biological sentinels, or ‘coastal canaries’ (Orth et al. 2006). For instance, eelgrass (*Zostera marina*) is included as a bio-indicator within the European Union water framework directive (Krause-Jensen et al. 2005). In tropical regions, most of the knowledge of temporal patterns and variability of seagrass distribution comes from Australia and the Caribbean (e.g. Rasheed and Unsworth 2011; Short et al. 2014; McKenzie et al. 2016). For large parts of the tropics, particularly in developing regions like East Africa, seagrasses are often forgotten or ignored in natural resource management (Unsworth and Cullen 2010) and knowledge about seagrass temporal variability is scarce (but see Gullström et al. 2006). Consequently, there is a great need to promote the development of seagrass monitoring programmes in such areas, both to provide baseline data for future assessments and to understand the effects of environmental change (Waycott et al. 2009; Nordlund et al. 2014; Cullen-Unsworth et al. 2014).

In this study, we investigated the temporal dynamics of a multispecific seagrass meadow, focusing on both intra- and inter-annual variation. We used data from a 10-year seagrass monitoring programme (SeagrassNet) in a marine-protected area in Zanzibar (Tanzania) to assess (i) if and how seagrass cover and species composition changed over time, (ii) to what extent different seagrass species changed over time, and (iii) if temporal variations in seagrass cover were linked to local environmental conditions.

## Materials and methods

### Study site

**Fig. 1:**
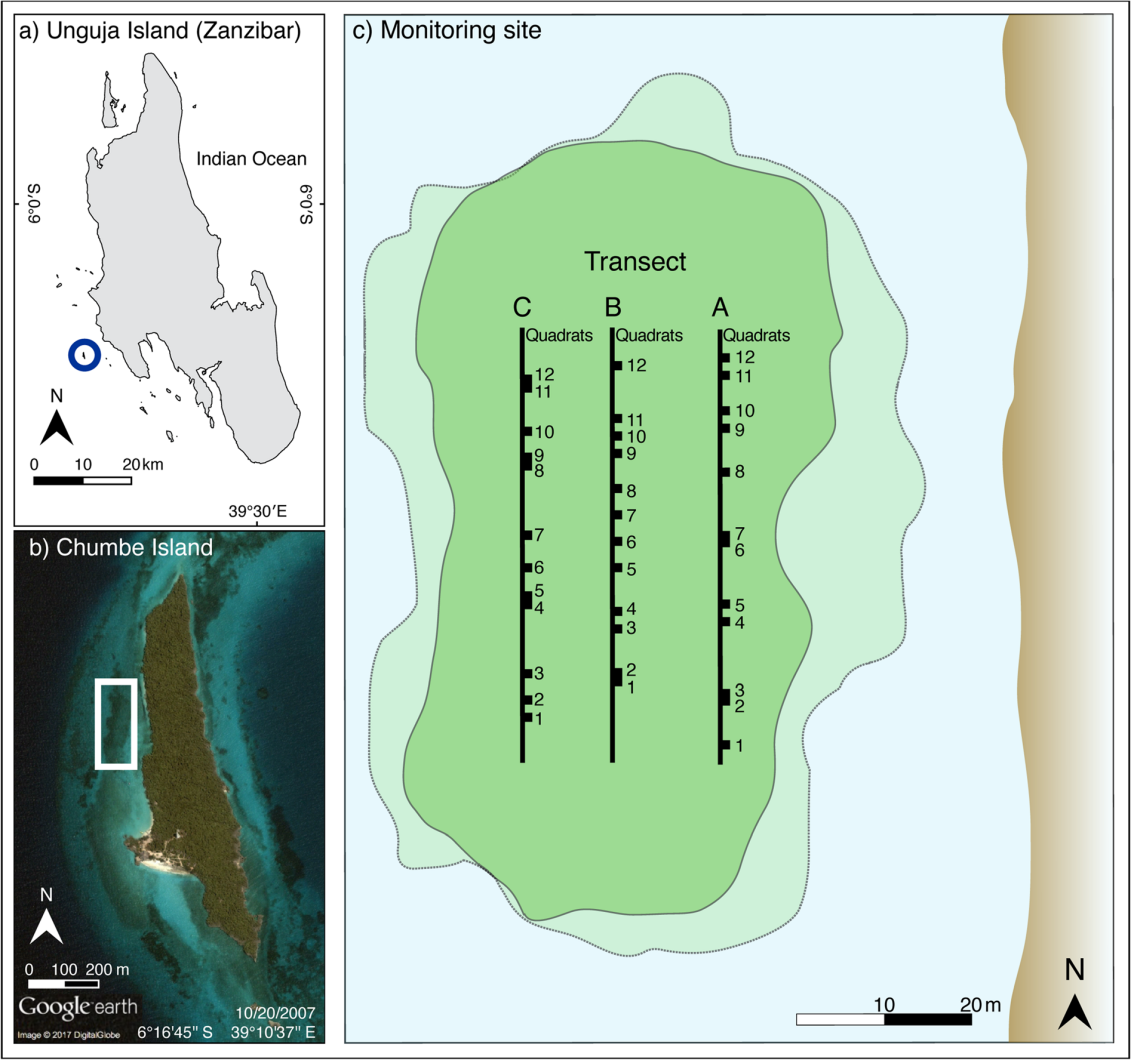
Map of the monitoring site. **a** Unguja Island (Zanzibar, Tanzania), where the circle marks the location of Chumbe Island. **b** Chumbe Island, where the white box marks the location of the monitoring site. **c** Depiction of the monitoring site with the three transects in the intertidal zone (A transect closest to shore; B mid-distance transect; C transect furthest from shore) and location of each of the 12 permanent quadrats

The seagrass monitoring site is located in Chumbe Island Coral Park (CHICOP); a strictly enforced no-take marine protected area (MPA). Chumbe Island is a small island (0.33 km^2^) located about 6 km west of Unguja Island (the larger island in Zanzibar), Tanzania (Fig. 1). CHICOP was established in 1991 and is since then privately managed (Nordlund et al. 2013). The MPA covers a 55.06 ha coral reef sanctuary (CRS) that hosts a diverse fringing coral reef and a scattered mosaic of seagrass meadows and patches. The seagrass species present in the CRS are *Cymodocea rotundata*, *Halodule* spp., *Thalassia hemprichii*, *Thalassodendron ciliatum*, *Halophila* spp., *Syringodium isoetifolium*, and *Cymodocea serrulata* (Knudby and Nordlund 2011; CHICOP 2017). The tidal regime in the region is semidiurnal, with an average tidal amplitude ranging from 3.2 m during spring tides and 0.9 m during neap tide (Shaghude et al. 2002).

In 2006, CHICOP established a global seagrass monitoring programme following the protocol of ‘SeagrassNet’ (www.seagrassnet.org). The monitoring site (TZ19.2, S 6° 16.6017′ E 39° 10.5733′, Fig. 1b) comprises three permanent transects (each 50 m long) placed parallel to the shore in the intertidal zone (Short et al. 2006b, Fig. 1c). Transects (A) closest to the shore is exposed most days of the tidal cycle, the middle transect (B) is exposed some days of the tidal cycle, while the transect placed furthest from shore (C) is only exposed during extreme spring low tides (Fig. 1c). All transects are sampled when exposed during spring low tide. Six seagrass species are present in the monitoring site: *C. serrulata*, *C. rotundata*, *Halodule* spp., *T. hemprichii*, *Halophila* spp., and *S. isoetifolium*.

### Data collection

#### Seagrass variables

The seagrass monitoring data were collected quarterly (four times per year) from 2007 to 2016, following the standardized SeagrassNet sampling protocol (Short et al. 2006b). No destructive sampling (i.e. seagrass voucher specimen and biomass samples) was conducted, since extractive activities are not allowed within the CRS according to the conservation status of CHICOP. In four occasions during the monitoring period, seagrass data were not collected (quarter 1 in 2010, quarters 1 and 4 in 2011, and quarter 3 in 2016). Along each transect, twelve quadrats (50 × 50 cm) were positioned using random distances provided by the SeagrassNet protocol. Each quadrat was first photographed from a vertical angle and then the following data were recorded: (1) species of seagrasses present, (2) visual estimation of total seagrass % cover, (3) visual estimation of seagrass cover of each species, (4) canopy height of the dominant species, and (5) shoot density (for large-sized species only) using a smaller quadrat (25 × 25 cm) positioned at the lower right corner of the larger quadrat. In this study, only seagrass cover data (overall and per species) was used.

#### Environmental variables

Seven environmental variables were chosen based on knowledge on their role as drivers of seagrass variability in tropical environments (Short et al. 2006a; Rasheed and Unsworth 2011; Rasheed et al. 2014; Marques et al. 2015; McKenzie et al. 2016). These variables were the following: air temperature (in lieu of water temperature), precipitation (rainfall), wind speed (as a proxy for wave action), cloudiness (% cloud cover), storm occurrence, number of sunspots, and low-tide exposure variables. Data on temperature, rainfall, and the speed and direction of winds were retrieved from NOAA/NCDC (https://www7.ncdc.noaa.gov/CDO/cdo) for the nearest weather station at Zanzibar airport (located ca. 8.5 km from the sampling site). Cloud cover data were obtained from the European Centre for Medium-Range Weather Forecasts (ECMWF) interim reanalysis (ERA-Interim; http://apps.ecmwf.int/datasets/, Dee et al. 2011) for the grid 39E/6S. Sunspot activity data was retrieved from the World Data Center SILSO (Royal Observatory of Belgium, Brussels, http://www.sidc.be/SILSO/). Tidal data was obtained from standard tide tables for Zanzibar harbour.

Three air temperature variables were considered: minimum, mean, and maximum daily temperatures (°C). Rainfall was calculated as the total daily rainfall (mm). Regarding wind speed, we first selected data on winds that are likely to create waves that would affect the study site, i.e. winds with directions of < 11° and > 180°. We then calculated the mean and maximum daily wind speeds (m/s). We also calculated a storm occurrence binary factor (1 = yes, 0 = no) indicating the occurrence of storms (maximum daily wind speed ≥ 24.5 m/s in any direction). Cloud cover was calculated as the average monthly total cloud cover (%). The number of sunspots is the total number of sunspots on each day. To assess the effects of low-tide exposure, we used two tidal variables: tidal amplitude and low tide height. The tidal amplitude represents the difference (in m) between the height of the diurnal low and high tides. Low tide height is the height of the diurnal low tide (in m).

Since seagrass monitoring took place approximately every 3 months, we calculated values for each environmental variable for the 1, 2, and 3 months previous to each monitoring event, yielding three variables per climate factor, and a total of 33 variables for input in the models (listed in ESM 2). Temperature, rainfall, wind, cloud cover, and number of sunspots variables were calculated as the average for the previous 1, 2, and 3 months. Storm occurrence (1/0) indicates the occurrence of storms during the previous 1, 2, and 3 months. Tidal variables were calculated as the maximum tidal amplitude and the minimum diurnal low tide height for the previous 1, 2, and 3 months.

### Statistical analyses

All statistical analyses were conducted using R v. 3.4.1. (R Core Team 2017). Response variables were checked for normality and homogeneity of variance before analyses. For all analyses, significance levels were set at α = 0.05.

#### Analyses of temporal changes in seagrass and environmental variables

To assess how seagrass cover changed during the 10-year study period, we first specified and tested a linear mixed-effects model with an interaction between year (factor) and transect (factor, 3 levels) as predictors of total seagrass cover. Year was treated as a factor to evaluate year-to-year changes. Thereafter, to test for potential changes in seasonal patterns of seagrass cover within each transect, a second linear mixed-effects model was run with an interaction between year (continuous), quarter of the year (factor, 4 levels), and transect (factor, 3 levels) as predictors of total seagrass cover. In these models, each measurement per quadrat and time point was used as a replicate, and quadrat was included as a random factor (random intercept only). To assess how the cover of each seagrass species changed during the study period, we run a linear mixed-effects model with seagrass cover as response variable and an interaction between species (factor, 6 levels), year (continuous), and transect (factor, 3 levels) as predictors. The model included quadrat as a random factor (random intercept) and a variance structure (varIdent) allowing for different variances for each species. Linear mixed-effects models were also used to evaluate temporal trends in each of the environmental variables (except storm occurrence) during the study period, with year as a continuous predictor and month as a random factor (random intercept only). When there was no temporal trend (i.e. the effect of year was not significant), we run linear mixed-effects models with year as a factor to evaluate potential non-linear changes.

Linear mixed-effects models were run using the “lme” function in the {nlme} R package (Pinheiro et al. 2017). Models were tested for possible temporal autocorrelation by plotting autocorrelation functions (ACF). When autocorrelation was present, an autocorrelation structure of order 1 (corAR1) was included in the models. Model fit was evaluated by likelihood ratio tests, comparing the models to the null models with only random effects. Model *R*^2^ were obtained using the “r.squaredGLMM” function from the {MuMIn} R package (Bartoń 2016). When a significant interaction between factors was included, we assessed the interaction through Tukey’s all-pair comparisons of means (“lsmeans” in {lsmeans}) (Lenth 2016). Tukey’s all-pair comparisons were also used to assess significance of differences between levels within a factor when it was a significant predictor in the model. When interactions occurred between a factor and a continuous variable, slopes were compared for each level of the factor using “lstrends” in {lsmeans} (Lenth 2016).

To evaluate the temporal changes in seagrass species composition (based on cover per species), we ran a constrained analysis of principal coordinates (CAP) with the “capscale” function from the {vegan} R package (Oksanen et al. 2016), based on Bray-Curtis dissimilarities and 999 permutations. An interaction between year (factor) and transect (factor) was included as predictor of the variability in the community composition. For this analysis, we used data at the transect level (i.e. averaging the data from all quadrats per transect per time point), yielding one replicate per transect and time point.

#### Predictors of seagrass cover change

To explore the effects of each of the environmental variables on seagrass cover, we ran linear mixed-effects models with the “lme” function in the {nlme} R package (Pinheiro et al. 2017). First, all environmental variables were tested for multicollinearity using pairwise Pearson’s correlation tests. Variables were considered to be too correlated if Pearson’s *R* was ≥ 0.6. Each climate variable seemed to be correlated with itself at different time frames, e.g. mean temperature for the previous month was correlated with mean temperature for the previous 2 and 3 months. Thus, only one timeframe per variable could be included in the models. To test which environmental variables significantly influenced seagrass cover overall (full model, i.e. merging data from all three transects) and per transect, we fitted models with each possible combination of predictors, assuring that no correlated predictors were included together in the models. Models were simplified through stepwise backwards removal of non-significant variables and compared using Akaike’s information criterion corrected for small sample sizes (AICc) (Burnham and Anderson 2002). A model was considered a better fit of the data when the delta AICc was > 4 units (Burnham and Anderson 2002). In these analyses, quadrat was included as a random factor (random intercept only), and data were standardized (scaled by means and standard deviations) to make coefficients comparable (Grace and Bollen 2005).

## Results

### Temporal changes in seagrass cover

**Fig. 2:**
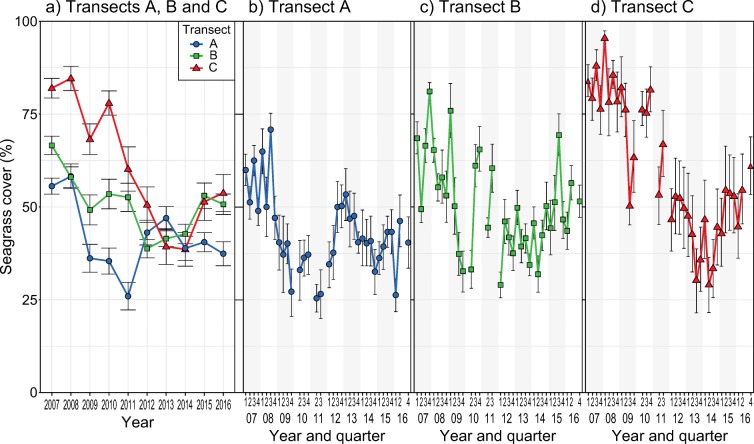
Changes in seagrass cover per **a** transect (A, B, and C) and year (2007–2016) and per quarter of the year for **b** transect A (closest to shore), **c** transect B, and **d** transect C. Each point represents the average cover of quadrats (*n* = 12) per year (**a**) and per quarter of the year (**b**-**d**). Error bars indicate standard deviation

There was high temporal variability in seagrass cover in all three transects throughout the 10-year study period. In short, the data showed a pattern of a gradual decline in total seagrass cover followed by a gradual increase; however, the timing of declines and increases was not synchronized across transects (Fig. 2a). The linear mixed-effects model on year-to-year changes of seagrass cover showed a significant interaction between year (factor) and transect (*P* < 0.001, Table S1 in ESM 1). Transects A (closest to shore) and B showed a decline in seagrass cover from 2007 (i.e. the beginning of the time series) until 2011 and 2012, respectively. While the yearly means in seagrass cover for 2010 and 2011 may be underestimated due to missing data in some quarters, the pattern of decline is still evident, particularly during 2008–2009 when the strongest decline in seagrass cover in transect A occurred. Transect C (furthest from shore), on the other hand, suffered a slightly stronger and longer period of decline in total cover, changing from an average seagrass cover of 82% in 2007, the highest of all transects, to 39% cover in 2014. At the end of the study period (last 2–4 years), seagrass cover increased slightly in all three transects reaching cover values of 37%, 51%, and 54% in transects A, B, and C, respectively, by 2016.

The linear mixed-effects model investigating seasonality patterns of total seagrass cover for each transect showed a significant interactive effect of transect, year, and quarter of the year (Table 1, Figs. 2b-d). In general, the total seagrass cover declined the most in quarter 1 (January–March) in all three transects. Transects A and B also suffered declines during quarter 3 (April–June) and 4 (October–December), respectively, while transect C showed declines in seagrass cover throughout the year. These differential patterns of decline led to changes in seasonality patterns (Fig. 2b-d). As in the previous analysis, transect C showed the strongest decline during the study period (2007–2016).

**Table 1:** Results from linear mixed-effect model assessing temporal and seasonal changes in seagrass cover per transect

**Response**	**Predictors**				***F*-statistic**	***P*** **value**	***R***^**2**^_**marginal**_	***R***^**2**^_**conditional**_	
Seagrass cover							0.182	0.446	
	Year (continuous)				66.15	**< 0.001**			
	Transect				4.00	0.278			
	Quarter of the year				9.79	**< 0.001**			
	Year (continuous) × transect				8.69	**< 0.001**			
	Year (continuous) × quarter of the year				14.97	**< 0.001**			
	Transect × quarter of the year				2.55	**0.019**			
	Year (continuous) × transect × quarter of the year				4.21	**< 0.001**			
**Effect of year (continuous) per quarter of the year and transect:**									
	**Transect A**			**Transect B**			**Transect C**		
Quarter of the year	Estimate	SE	*P value*	Estimate	SE	*P value*	Estimate	SE	*P value*
Quarter 1	**− 2.734**	0.666	**< 0.001**	**− 3.035**	0.673	**< 0.001**	**− 5.670**	0.673	**< 0.001**
Quarter 2	− 0.357	0.675	0.596	− 0.322	0.680	0.636	**− 4.266**	0.680	**< 0.001**
Quarter 3	**− 2.319**	0.675	**< 0.001**	− 0.185	0.784	0.805	**− 4.841**	0.748	**< 0.001**
Quarter 4	− 0.497	0.658	0.451	**− 2.253**	0.661	**< 0.001**	**− 3.238**	0.661	**< 0.001**

Values in bold indicate significant effects

*SE*, standard error; *R*^*2*^_*marginal*_,variance explained by the predictors; *R*^*2*^_*conditional*_, variance explained by both fixed (predictors) and random factors (quadrat)

**Fig. 3:**
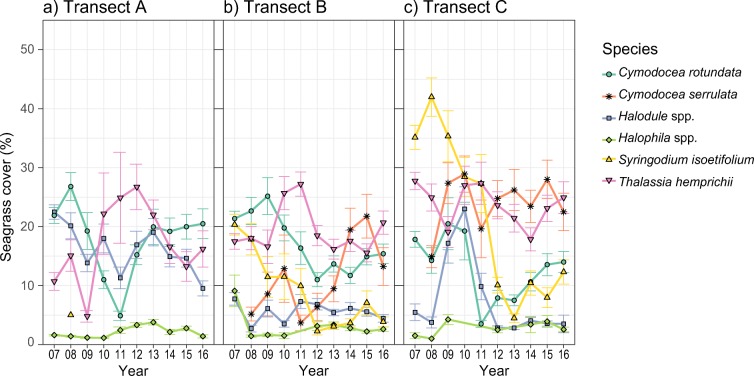
Changes in seagrass cover per species and year for **a** transect A, **b** transect B, and **c** transect C. Each point represents the average cover per species and year. Error bars indicate standard deviation

The model assessing species-specific responses revealed that temporal patterns in seagrass cover differed between the co-occurring seagrass species (Table 2S in ESM 1). Most species experienced a decline during the study period, although not in all transects. *Syringodium isoetifolium* declined in all transects where it was present (transects B and C, *P* < 0.001). Other species declined in some transects while remaining stable in the rest: *Cymodocea rotundata* (declined in transect B, *P* = 0.0219), *Thalassia hemprichii* (declined in transects A and C, *P* < 0.001), *Halodule* spp. (declined in transect A, *P* = 0.0152), and *Halophila* spp. (declined in transect A, *P* = 0.041). Contrarily, *Cymodocea serrulata* increased in cover in two transects (transects B and C, *P* < 0.001), while remaining stable in transect A. Year-to-year changes in cover per species (Fig. 3) illustrate a high temporal variability, and patterns of replacement are observed. For instance, declines in *C. rotundata* are followed by increases in *T. hemprichii*, while increases in *C. serrulata* follow declines in *S. isoetifolium* (Fig. 3).

### Temporal changes in seagrass species composition

**Fig. 4:**
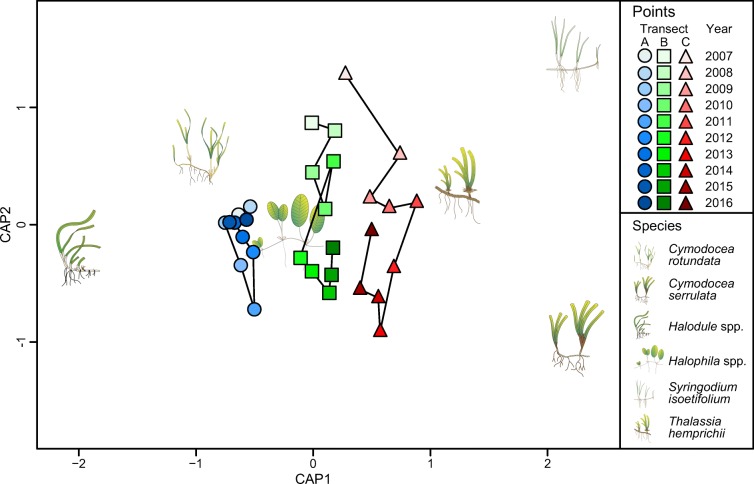
Results from the constrained ordination analysis (CAP) displaying the changes in species composition per transect and year. Each point represents the average species composition per transect and year. Lines connect consecutive years per transect. Symbols illustrate the position of each seagrass species in the multivariate space. Seagrass symbols: Catherine Collier and the Integration and Application Network, University of Maryland Center for Environmental Science (ian.umces.edu/symbols/)

There was a significant interactive effect of year and transect on seagrass species composition (based on cover per species) (CAP; *F* = 14.31, *P* = 0.001). Similarly to seagrass cover, the analysis of species composition displayed a pattern of change and recovery (Fig. 4). Year-to-year trajectories showed a clear departure from the initial species composition in all three transects. In transect A, the departure from 2007 was followed by a recovery period from 2011, returning to the initial species composition by 2016. In transects B and C, on the other hand, the recovery pattern seemed to have started later, with year-to-year trajectories moving towards the origin from 2013 and 2014, respectively, but not (yet) reaching the initial species composition.

### Temporal changes in environmental variables

Most of the environmental variables changed during the study period (Fig. 1S in ESM 1). First, linear mixed-effects models revealed that the mean, minimum, and maximum air temperatures increased over time by 0.07 °C (*P* < 0.001), 0.10 °C (*P* < 0.001), and 0.05 °C, respectively (*P* = 0.040), while the mean wind speed increased over time by 0.04 m/s (*P* = 0.017). The height of the diurnal low tide decreased over time by 0.09 m (*P* < 0.001), and the tidal amplitude increased by 0.16 m (*P* < 0.001), potentially due to cyclical changes in tidal amplitude (Haigh et al. 2011). This means that the diurnal low tides got lower, increasing seagrass exposure to air. Maximum wind speed, rainfall, sunspot activity, and cloud cover did not show significant trends during the study period (*P* > 0.05). However, in the models that included year as a categorical factor, maximum wind speed increased from 2009 until 2012 and then decreased until 2015 (*P* < 0.001), while sunspot activity increased from 2007 until 2014 and then decreased until 2016 (*P* < 0.001).

### Predictors of seagrass cover change

**Fig. 5:**
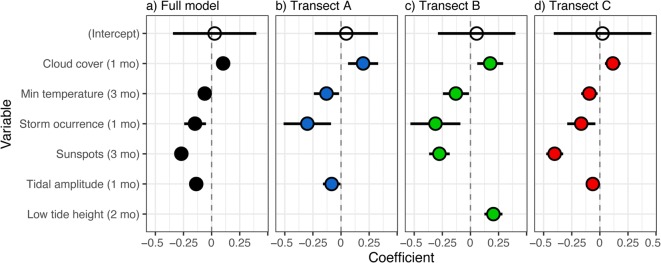
Results from the linear mixed-effects models assessing the influence of environmental variables on seagrass cover in **a** all three transects, **b** transect A, **c** transect B, and **d** transect C, estimated as partial regression coefficients. Variables included the following: cloud cover (1 month) = mean cloud cover of the previous month; min. temperature (3 months) = mean minimum daily air temperature of the previous 3 months; storm occurrence (1 month) = storm occurrence during the previous month; sunspots (3 months) = sunspot activity during the previous 3 months; tidal amplitude (1 month) = maximum tidal amplitude during the previous month; and low tide height (2 months) = minimum height of the diurnal low-tide during the previous 2 months. Points represent the standardized coefficients ± 95% confidence intervals (black bars). Confidence intervals that do not overlap zero indicate significant standardized coefficients (illustrated as filled points)

Out of the 33 environmental sub-variables tested (listed in ESM 2), six appeared to influence seagrass cover: cloudiness, minimum temperature, storm occurrence, sunspot activity, tidal amplitude, and the height of the diurnal low tide. However, not all variables were significant at all times in the models (Fig. 5 and Fig. 2S in ESM 1). Three variables where significant in all models tested (full model and models for transects A, B, and C): occurrence of storms during the previous month (*P* = 0.003, 0.005, 0.006, and 0.009, respectively) and minimum temperature during the previous 3 months (*P = 0.032*, 0.041, 0.029, and 0.012, respectively) decreased seagrass cover, while cloudiness during the previous month increased the cover (*P* < 0.001 and *P* = 0.004, 0.003, and 0.001, respectively). Maximum tidal amplitude during the previous month had a negative effect on the full model and models for transects A and C (*P* < 0.001 and *P* = 0.035 and 0.006, respectively), while the minimum height of the diurnal low tide during the previous 2 months had a positive effect in transect B (*P* < 0.001). Sunspot activity during the previous 3 months reduced seagrass cover in transects B and C, as well as in the full model (*P* < 0.001).

Even though these variables appeared to influence seagrass cover, the variance explained by the full model, as well as the models for each individual transect, was low (*R*^2^_marginal_ = 0.11, 0.03, 0.13, and 0.19, for the full model and models for transects A, B and C, respectively, where *R*^2^_marginal_ is the variance explained by the fixed variables). Thus, environmental variables had a significant but small effect on seagrass cover during the study period. When taking into account also the random factor included in the model (quadrat), the variance explained greatly increases in most models (*R*^2^_conditional_ = 0.50, 0.05, 0.34, and 0.69 for the full model and models for transects A, B and C, respectively, where *R*^2^_conditional_ is the variance explained by both fixed and random factors).

## Discussion

Our results show a clear gradual decline in seagrass cover (of up to 50%) and a shift in species composition, followed by a gradual recovery in both cover and species composition, over the 10-year period studied. These temporal patterns differed between the three transects in both timing and length. Moreover, the observed temporal changes could only partly be related to changes in environmental variables, suggesting that other factors may be more important for the observed seagrass dynamics. Our study highlights the challenges of designing a monitoring programme and which variables to measure and analyse to understand the causes of change.

To our knowledge, there has only been one other study evaluating seagrass temporal dynamics in the East African region (Gullström et al. 2006). In that study, the authors used satellite remote sensing to assess changes in large-scale areal cover of seagrass within a bay over a 16-year period (comparing two time-points; 1987 to 2003). The total area with seagrass did not change, although both losses and gains were observed in different areas. Localized patterns of decline and recovery of seagrass cover, such as the ones found in the present study, could help to explain the overall stability observed over longer timeframes and across spatial scales. Unlike our study, Gullström et al. (2006) found no seasonal changes in seagrass cover. This contradiction could potentially be explained by the differences in spatial scale and depth environments. Seasonal changes in seagrass cover observed in intertidal areas at small spatial scales (10–50 m, our study) may not be detected when averaged over both inter and subtidal areas and at larger spatial scales (several km, Gullström et al. 2006), potentially due to species-specific seasonality (Duarte et al. 2006) and across depth zones (Meling-López and Ibarra-Obando 1999). Previous monitoring studies on tropical seagrass meadows in other regions have shown a variety of temporal patterns in seagrass cover, from strong declines to some cases of increases (Short et al. 2006a; López-Calderón et al. 2013; Yaakub et al. 2014; McKenzie et al. 2016; Wynne 2017; Qiu et al. 2017), even though decline seems to be the most common trend (Short et al. 2006a; Freeman et al. 2008; Unsworth et al. 2012; Rasheed et al. 2014; Chen et al. 2016). Most studies, however, analysed relatively short time series (< 8 years). Our data also showed a pattern of decline during the first 4 to 7 years (depending on transect), but this reduction was followed by a period of recovery. This illustrates that longer time scales (> 10 years) may be necessary to fully capture and better understand the dynamics of seagrass meadows.

Of the seven seagrass species described from Chumbe Island (Knudby and Nordlund 2011; CHICOP 2017), six were observed in the monitored transects (*Cymodocea rotundata*, *Cymodocea serrulata*, *Halodule* spp., *Halophila* spp., *Syringodium isoetifolium*, and *Thalassia hemprichii*). These make up about half of the species in the region (sensu Gullström et al. 2002, Duarte et al. 2012), and represent c. 10% of the world’s seagrasses (Green and Short 2003). Seagrass species composition changed throughout the study period in all three transects. We observed a gradual change in species composition. First, species composition deviated from the original composition (at the start of the study period, 2007). After a period of departure, a switch in the direction of change was detected from 2011, 2013, and 2014 in transects A, B, and C respectively, with species compositions moving back towards the 2007 composition. The observed changes in species composition were potentially caused by species-specific patterns. For instance, our data showed that while some species declined over time (e.g. *C. rotundata* and *S. isoetifolium*), others increased (*C. serrulata*). At the same time, certain species seemed to increase in cover following declines of another species (e.g. *T. hemprichii* and *C. serrulata* increases followed declines in *C. rotundata* and *S. isoetifolium*, respectively). Similar patterns of species replacement have been observed in previous studies (e.g. Short et al. 2014; Wynne 2017). These patterns could be explained by two mechanisms. First, different species may respond differently to external pressures, such as anthropogenic or climate impacts; for instance, certain seagrass species, such as *C. serrulata*, are more tolerant to high temperatures than others, e.g. *S. isoetifolium* (Campbell et al. 2006). At the same time, species may also respond differently during the recovery process after disturbance, with some species recovering faster than others (Rasheed et al. 2014; Nowicki et al. 2017). Secondly, when some seagrass species decline, there is a release of available space and a decrease in competition, which may stimulate growth and colonization by less competitive, opportunist species. Thus, multispecific seagrass meadows may be more resilient to external impacts than monospecific meadows, by partially buffering seagrass decline.

We investigated whether the observed changes in seagrass cover could be explained by changes in environmental variables. Of all the environmental variables considered, only three seemed to influence seagrass cover across the three transects: storm occurrence, minimum temperature, and cloudiness. The occurrence of storms had a negative impact on seagrass cover, concurring with previous studies (Short et al. 2006a; Rasheed et al. 2014, but see van Tussenbroek et al. 2014), which may be explained by increased water turbidity, reducing light availability for seagrasses (Rasheed et al. 2014); sediment movement, causing plant burial (Short et al. 2006a); as well as water movement, causing plant uprooting (Rasheed et al. 2014). Minimum temperature also had a negative influence on seagrass cover. Previous studies have also shown negative effects of increased temperature on tropical seagrass meadows (Rasheed and Unsworth 2011; McKenzie et al. 2016), although other studies have shown positive (López-Calderón et al. 2013) or neutral effects of temperature on seagrasses (Freeman et al. 2008; Qiu et al. 2017). Even though extreme temperatures (40–45 °C) may adversely impact seagrasses (Campbell et al. 2006; Koch et al. 2013, George et al. 2018), moderate temperature increases may actually prove beneficial (Koch et al. 2013). In addition, tropical seagrasses can maintain unaffected photosynthetic capacity during high temperature stress, while simultaneously suffering from biomass loss (George et al. 2018). The fact that minimum temperature was the only temperature factor that seemed to influence seagrass cover could be due to the stronger changes in minimum temperature during the study period, while mean and maximum temperatures varied less. Cloudiness, on the other hand, was found to positively affect seagrass cover, in agreement with previous studies (e.g. McKenzie et al. 2016), potentially due to a reduced impact of direct sun exposure on shallow seagrasses during low tide periods.

Three other environmental variables influenced seagrass cover in some but not all transects. Tidal amplitude had a negative effect on seagrass cover overall and in transects A and C, while the height of the diurnal low tide had a positive influence on seagrass cover in transect B, i.e. the higher the water was during low tide, the higher the seagrass cover; or rather, lower water level in shallow areas during low tide had a negative impact on seagrass cover. Previous studies have shown contradictory results regarding the effects of tidal exposure on seagrass cover, showing both positive (Rasheed and Unsworth 2011; McKenzie et al. 2016) and negative effects (Erftemeijer and Herman 1994; Unsworth et al. 2012). While lower water may cause exposure of seagrasses to air and direct sunlight, leading to high temperatures, burning, and desiccation (Björk et al. 1999), periods of lower water may also provide a critical window of sufficient light for seagrass photosynthetic processes (Pollard and Greenway 1993). As the monitoring site in the present study consists of a shallow intertidal seagrass meadow, tidal exposure is more likely to have a negative effect on seagrasses at this site, compared with areas more distant to the shore. Moreover, the finding that the effect of tidal amplitude was weaker in the transect furthest from shore (transect C) suggests that deeper areas may act as a refuge from extreme low-water events. Sunspot activity showed a negative effect on seagrass cover overall and in transects B and C, but not in transect A. To date, only two studies have evaluated the influence of sunspot activity on seagrasses. First, solar activity seems to be the main trigger of massive flowering events in *Posidonia oceanica* meadows; a common stress response in plants (Montefalcone et al. 2013). Second, in a tropical *Halodule wrightii* meadow, seagrass canopy height and shoot density were negatively related to solar activity at high sunspots numbers (Marques et al. 2015). It should also be noted that solar radiation has been shown to negatively affect seagrass cover (Unsworth et al. 2012).

Even though the above-mentioned variables appear to all influence seagrass cover, they together explained quite a small portion of the observed variability. This might be due to the relatively short time period studied. Previous studies have also found that 10 years of data may not be enough to fully explain the sources of seagrass variability (e.g. Yaakub et al. 2014); however, other studies have been able to detect strong correlations between environmental variables and seagrass cover at similar or even shorter temporal scales than in the present study (8–11 years) (Unsworth et al. 2012; López-Calderón et al. 2013; Marques et al. 2015; McKenzie et al. 2016). This suggests that other, unmeasured variables may be responsible for the observed pattern of seagrass decline and recovery at Chumbe. For instance, alterations in water currents due to changes in wind patterns or morphology of the sea bottom could have caused loss of seagrass through erosion or burial due to changes in sediment distribution (Cabaço et al. 2008). A previous study at the Chumbe reef found strong seasonal sediment fluxes during the southeast monsoon season, due to altered water currents and wind patterns (Muzuka et al. 2010). Such increases in sediment-driven turbidity could potentially also influence the nearby seagrass meadows. It is also possible that the observed changes in seagrass cover and species composition were caused by natural shifts throughout the site, which could even be part of longer temporal dynamics. However, given the lack of data available regarding these and other potential drivers, we cannot evaluate their role in controlling seagrass cover at the study site. The lack of a clear cause for seagrass loss also makes it difficult to assess potential drivers of recovery. Given the species-specific patterns of change observed, the multispecific nature of the seagrass meadow may have potentially played a role in both reducing the impact of seagrass loss and enhancing recovery.

In conclusion, the studied seagrass meadow appears to be highly dynamic, with observed changes in cover and species composition across seasons and between years. Considering that the study site is formally protected and that no major developments have occurred in the area (Nordlund et al. 2013), seagrass meadows can be seen as highly dynamic ecosystems even in the absence of major local impacts. In fact, our findings suggest that seagrass variability is highly localized, which is supported by the variability explained by differences between transects (located < 50 m apart) and even between quadrats (located < 10 m apart). The observed changes in seagrass cover could only weakly be explained by the influence of local environmental variables. High-resolution monitoring programmes, such as the one described in the present study, seem highly valuable for detection of temporal changes in seagrass cover within complex multispecific seagrass habitats. However, to understand the causes for the patterns observed, our findings confirm the need for long-term (> 10 years) data series that include direct measurements of potential variables influencing seagrasses at the considered site (e.g. water temperature, light availability, salinity, sediment movement, and turbidity), as well as unusual or extreme events (e.g. massive storms and heat waves).

## Electronic supplementary material

ESM 1 (PDF 4086 kb)

ESM 2 (PDF 63 kb)
