# Supplementary material for: Temporal variability of a protected multispecific tropical seagrass meadow in response to environmental change
Source: Environ Monit Assess. 2019 Nov 26;191(12):774. doi: 10.1007/s10661-019-7977-z (PMC6879446; doi:10.1007/s10661-019-7977-z)
Supplement: Supplementary file 1 — (PDF 4086 kb) [file 10661_2019_7977_MOESM1_ESM.pdf]

Electronic Supplementary Material 1 (ESM1)

Article title
Temporal variability of a protected multispecific tropical seagrass meadow in response to environmental change

Journal
Environmental Monitoring and Assessment

Authors
E Alonso Aller, JS Eklöf, M Gullström, U Kloiber, HW Linderholm, LM Nordlund*

*Corresponding author
Natural Resources and Sustainable Development, Department of Earth Sciences, Uppsala University, Uppsala, Sweden
Email: lina.mtwana.nordlund@geo.uu.se

Table 1S Results from linear mixed-effect model and Tukey's all-pair comparisons of means for the model assessing the temporal changes in seagrass cover per transect

**Response**	**Predictors**	***F*-statistic**	***P*-value**	$R^2_{marginal}$	$R^2_{conditional}$
Seagrass cover				0.248	0.545
	Year (factor)	37.82	**< 0.001**		
	Transect	3.84	**0.021**		
	Year (factor) × Transect	9.85	**< 0.001**		

Tukey's all pair comparisons:

	**Transect A**			**Transect B**			**Transect C**		
Year to year comparisons	Estimate	SE	*P-value*	Estimate	SE	*P-value*	Estimate	SE	*P-value*
2007 - 2008	0.097	0.140	9.999	-0.314	0.140	0.428	0.096	0.140	9.999
2007 - 2009	**-0.713**	0.140	**< 0.001**	**-0.638**	0.140	**< 0.001**	**-0.505**	0.140	**0.012**
2007 - 2010	**-0.716**	0.151	**< 0.001**	-0.457	0.151	0.078	-0.123	0.151	0.998
2007 - 2011	**-1.072**	0.172	**< 0.001**	-0.498	0.172	0.110	**-0.784**	0.172	**< 0.001**
2007 - 2012	**-0.459**	0.140	**0.035**	**-1.019**	0.140	**< 0.001**	**-1.152**	0.140	**< 0.001**
2007 - 2013	-0.315	0.140	0.421	**-0.921**	0.140	**< 0.001**	**-1.566**	0.140	**< 0.001**
2007 - 2014	**-0.614**	0.140	**< 0.001**	**-0.876**	0.140	**< 0.001**	**-1.593**	0.140	**< 0.001**
2007 - 2015	**-0.553**	0.140	**0.003**	**-0.497**	0.140	**0.014**	**-1.123**	0.140	**< 0.001**
2007 - 2016	**-0.668**	0.140	**< 0.001**	**-0.564**	0.151	**0.008**	**-1.018**	0.151	**< 0.001**
2008 - 2009	**-0.810**	0.140	**< 0.001**	-0.324	0.140	0.377	**-0.601**	0.140	**< 0.001**
2008 - 2010	**-0.814**	0.151	**< 0.001**	-0.143	0.151	0.995	-0.220	0.151	0.911
2008 - 2011	**-1.169**	0.172	**< 0.001**	-0.184	0.172	0.988	**-0.880**	0.172	**< 0.001**
2008 - 2012	**-0.556**	0.140	**0.003**	**-0.706**	0.140	**< 0.001**	**-1.249**	0.140	**< 0.001**
2008 - 2013	-0.412	0.140	0.094	**-0.608**	0.140	**< 0.001**	**-1.662**	0.140	**< 0.001**
2008 - 2014	**-0.711**	0.140	**< 0.001**	**-0.562**	0.140	**0.002**	**-1.689**	0.140	**< 0.001**
2008 - 2015	**-0.650**	0.140	**< 0.001**	-0.184	0.140	0.951	**-1.220**	0.140	**< 0.001**
2008 - 2016	**-0.765**	0.140	**< 0.001**	-0.250	0.151	0.821	**-1.114**	0.151	**< 0.001**
2009 - 2010	-0.004	0.151	9.999	0.181	0.151	0.973	0.381	0.151	0.260
2009 - 2011	-0.359	0.172	0.540	0.140	0.172	0.998	-0.279	0.172	0.840
2009 - 2012	0.254	0.140	0.725	-0.382	0.140	0.163	**-0.648**	0.140	**< 0.001**
2009 - 2013	0.398	0.140	0.123	-0.284	0.140	0.579	**-1.061**	0.140	**< 0.001**
2009 - 2014	0.099	0.140	9.999	-0.238	0.140	0.795	**-1.088**	0.140	**< 0.001**
2009 - 2015	0.160	0.140	0.980	0.141	0.140	0.992	**-0.619**	0.140	**< 0.001**
2009 - 2016	0.045	0.140	9.999	0.074	0.151	9.999	**-0.513**	0.151	**0.025**
2010 - 2011	-0.355	0.181	0.627	-0.041	0.181	9.999	**-0.660**	0.181	**0.010**
2010 - 2012	0.258	0.151	0.795	**-0.563**	0.151	**0.008**	**-1.029**	0.151	**< 0.001**
2010 - 2013	0.401	0.151	0.196	-0.465	0.151	0.067	**-1.443**	0.151	**< 0.001**
2010 - 2014	0.102	0.151	9.999	-0.419	0.151	0.150	**-1.469**	0.151	**< 0.001**
2010 - 2015	0.164	0.151	0.987	-0.040	0.151	9.999	**-1.000**	0.151	**< 0.001**
2010 - 2016	0.049	0.151	9.999	-0.107	0.163	9.999	**-0.894**	0.163	**< 0.001**
2011 - 2012	**0.613**	0.172	**0.014**	-0.522	0.172	0.076	-0.369	0.172	0.500
2011 - 2013	**0.757**	0.172	**< 0.001**	-0.424	0.172	0.292	**-0.783**	0.172	**< 0.001**
2011 - 2014	0.458	0.172	0.193	-0.378	0.172	0.462	**-0.809**	0.172	**< 0.001**
2011 - 2015	0.519	0.172	0.079	0.001	0.172	9.999	-0.340	0.172	0.620
2011 - 2016	0.404	0.172	0.361	-0.066	0.183	9.999	-0.234	0.183	0.958
2012 - 2013	0.144	0.140	0.991	0.098	0.140	9.999	-0.414	0.140	0.091
2012 - 2014	-0.155	0.140	0.984	0.144	0.140	0.991	-0.440	0.140	0.053
2012 - 2015	-0.094	0.140	9.999	**0.522**	0.140	**0.007**	0.029	0.140	9.999
2012 - 2016	-0.209	0.140	0.895	0.455	0.151	0.080	0.135	0.151	0.997
2013 - 2014	-0.299	0.140	0.501	0.046	0.140	9.999	-0.027	0.140	9.999
2013 - 2015	-0.238	0.140	0.795	0.424	0.140	0.074	0.443	0.140	0.050
2013 - 2016	-0.353	0.140	0.258	0.358	0.151	0.351	**0.548**	0.151	**0.011**
2014 - 2015	0.061	0.140	9.999	0.379	0.140	0.172	**0.470**	0.140	**0.028**
2014 - 2016	-0.054	0.140	9.999	0.312	0.151	0.558	**0.575**	0.151	**0.006**
2015 - 2016	-0.115	0.140	0.998	-0.067	0.151	9.999	0.106	0.151	9.999

SE = Standard error. Values in bold indicate significant differences. $R^2_{marginal}$ = variance explained by the predictors. $R^2_{conditional}$ = variance explained by both fixed (predictors) and random factors (quadrat)

Table 2S Results from linear mixed-effect model assessing temporal and seasonal changes in seagrass cover per species

**Response**	**Predictors**	***F*-statistic**	***P*-value**	$R^2_{marginal}$	$R^2_{conditional}$
Seagrass cover				0.461	0.487
	Year (continuous)	23.24	**< 0.001**		
	Transect	4.63	**0.017**		
	Species	417.28	**< 0.001**		
	Year (continuous) × Transect	3.764	**0.023**		
	Year (continuous) × Species	35.31	**< 0.001**		
	Transect × Species	115.18	**< 0.001**		
	Year (continuous) × Transect × Species	11.29	**< 0.001**		

Effect of Year (continuous) per species and transect:

	**Transect A**			**Transect B**			**Transect C**		
Species	Estimate	SE	*P-value*	Estimate	SE	*P-value*	Estimate	SE	*P-value*
Cymodocea rotundata	-0.029	0.023	0.209	**-0.054**	0.023	**0.022**	-0.007	0.023	0.764
Cymodocea serrulata	0.006	0.021	0.762	**0.147**	0.021	**< 0.001**	**0.135**	0.021	**< 0.001**
Halodule spp.	**-0.053**	0.022	**0.015**	0.011	0.022	0.638	0.016	0.022	0.475
Halophila spp.	**-0.051**	0.017	**0.041**	-0.010	0.017	0.566	0.022	0.017	0.208
Thalassia hemprichii	**-0.081**	0.023	**< 0.001**	0.012	0.023	0.599	**-0.101**	0.023	**< 0.001**
Syringodium isoetifolium	-0.011	0.021	0.596	**-0.135**	0.021	**< 0.001**	**-0.258**	0.021	**< 0.001**

SE = Standard error. Values in bold indicate significant effects. $R^2_{marginal}$ = variance explained by the predictors. $R^2_{conditional}$ = variance explained by both fixed (predictors) and random factors (quadrat)

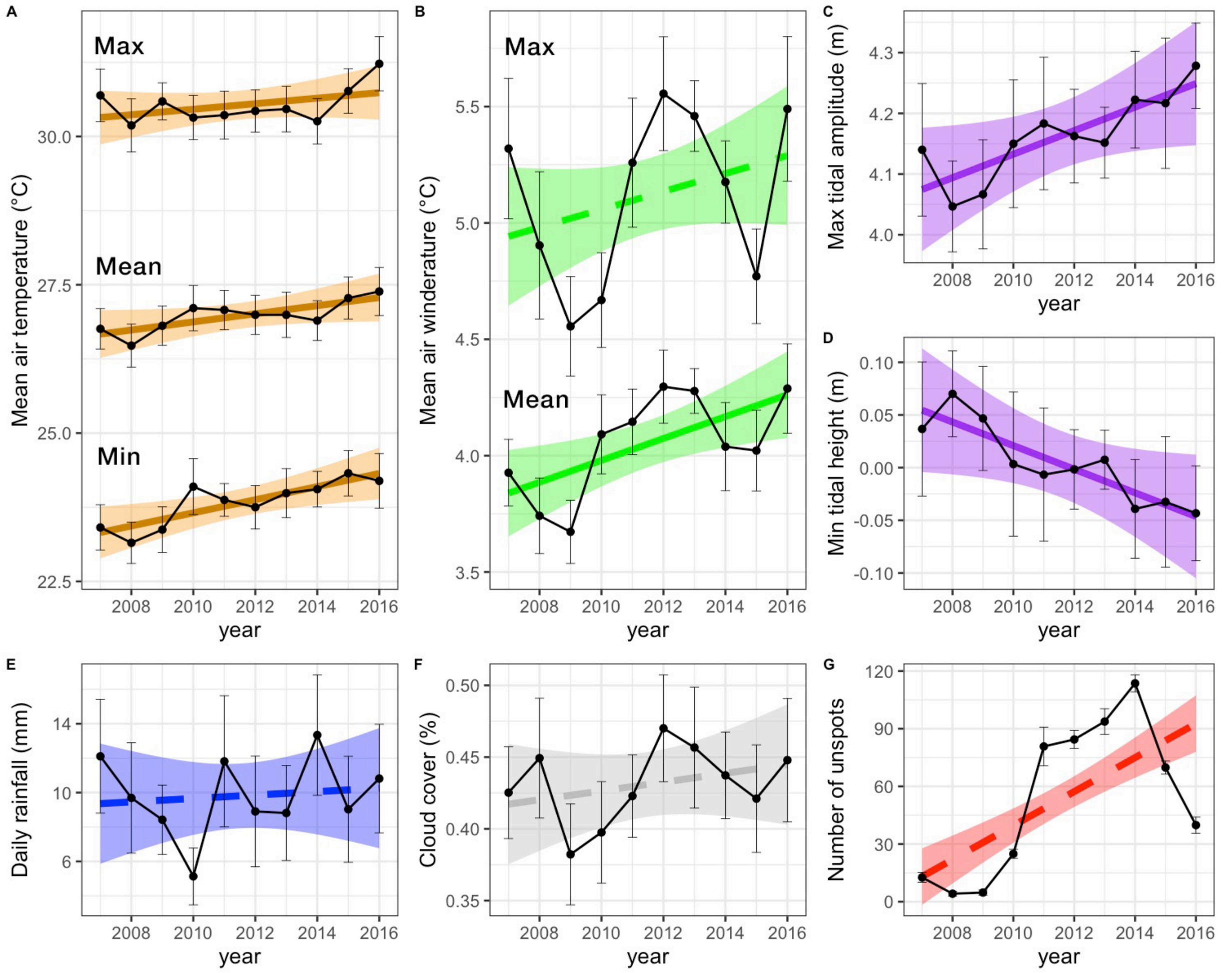

Fig. 1S Changes over time in average daily maximum, mean and minimum air temperature (A); average daily maximum and mean wind speed (B); maximum daily tidal amplitude (C); minimum daily height of the diurnal low tide (D); average total daily rainfall (E); monthly mean cloud cover (F); and average daily number of sunspots (G). Points represent the average value per year ± SD. Lines represent the conditional effect plots for the effect of year on each variable. Dashed lines represent non-significant changes. Shaded areas are 95% confidence intervals

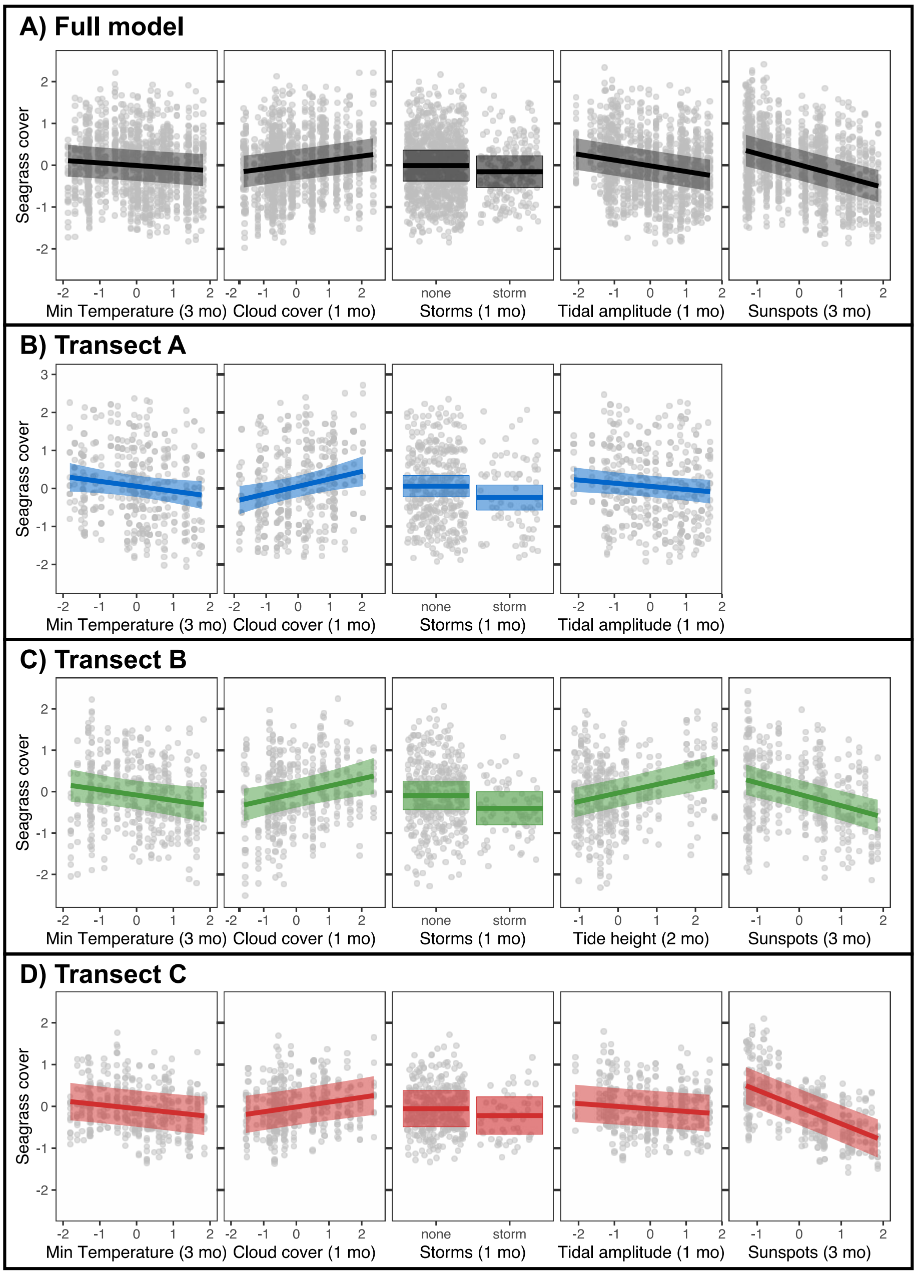

Fig. 2S Conditional effect plots for the predictors of seagrass cover in a) all three transects, b) transect A, c) transect B, and d) transect C. Min Temperature (3 mo) = average daily minimum temperature during the previous 3 months; Cloud cover (1mo) = mean cloud cover during the previous month; Storms (1 mo) = storm occurrence during the previous month; Tidal amplitude (1 mo) = maximum tidal amplitude during de previous month; Tide height (2 mo) = minimum height of the diurnal low tide during the previous 2 months; Sunspots (3 mo) = average daily number of sunspots during the previous 3 months. Lines represent the conditional effect plots for the effect of each variable on seagrass cover. Shaded areas are 95% confidence intervals. Grey dots are partial residuals
