# Supplementary material for: Temporal variability of a protected multispecific tropical seagrass meadow in response to environmental change
Source: Environ Monit Assess. 2019 Nov 26;191(12):774. doi: 10.1007/s10661-019-7977-z (PMC6879446; doi:10.1007/s10661-019-7977-z)
Supplement: Supplementary file 2 — (PDF 63 kb) [file 10661_2019_7977_MOESM2_ESM.pdf]

Electronic Supplementary Material 2 (ESM2)

Article title
Temporal variability of a protected multispecific tropical seagrass meadow in response to environmental change

Journal
Environmental Monitoring and Assessment

Authors
E Alonso Aller, JS Eklöf, M Gullström, U Kloiber, HW Linderholm, LM Nordlund*

*Corresponding author:
Natural Resources and Sustainable Development, Department of Earth Sciences, Uppsala University, Uppsala, Sweden
Email: lina.mtwana.nordlund@geo.uu.se

List of climate variables tested as predictors of change in seagrass cover

Average daily mean temperature during the previous month
Average daily mean temperature during the previous 2 months
Average daily mean temperature during the previous 3 months

Average daily minimum temperature during the previous month
Average daily minimum temperature during the previous 2 months
Average daily minimum temperature during the previous 3 months

Average daily maximum temperature during the previous month
Average daily maximum temperature during the previous 2 months
Average daily maximum temperature during the previous 3 months

Average total daily rainfall during the previous month
Average total daily rainfall during the previous 2 months
Average total daily rainfall during the previous 3 months

Average daily mean wind speed during the previous month
Average daily mean wind speed during the previous 2 months
Average daily mean wind speed during the previous 3 months

Average daily maximum wind speed during the previous month
Average daily maximum wind speed during the previous 2 months
Average daily maximum wind speed during the previous 3 months

Average monthly cloud cover during the previous month
Average monthly cloud cover during the previous 2 months
Average monthly cloud cover during the previous 3 months

Occurrence of storms during the previous month
Occurrence of storms during the previous 2 months
Occurrence of storms during the previous 3 months

Average daily number of sunspots during the previous month
Average daily number of sunspots during the previous 2 months
Average daily number of sunspots during the previous 3 months

Maximum tidal amplitude during the previous month
Maximum tidal amplitude during the previous 2 months
Maximum tidal amplitude during the previous 3 months

Minimum diurnal low tide height during the previous month
Minimum diurnal low tide height during the previous 2 months
Minimum diurnal low tide height during the previous 3 months
